# Pilot Study to Evaluate the Appropriate Management of Patients With Coexistent Bacterial Vaginosis and Cervicitis

**DOI:** 10.1155/S1064744995000445

**Published:** 1995

**Authors:** Jane R. Schwebke, Mary Beth Schulien, Mark Zajackowski

**Affiliations:** 1 Chicago Department of Health Chicago IL USA; 2 Medicine/Infectious Diseases Northwestern University Medical School Chicago IL USA; 3 University of Alabama at Birmingham 229 Tinsley Harrison Tower 1900 University Boulevard Birmingham AL 35294-0006 USA

## Abstract

A pilot study was performed to obtain preliminary data regarding the need for targeted therapy of bacterial vaginosis (BV) when it accompanies clinically defined cervicitis. Specifically, women attending a sexually transmitted disease (STD) clinic with clinically defined BV and cervicitis were treated in the first phase of the study with doxycycline alone. In phase II, the patients received doxycycline and concomitant intravaginal metronidazole gel. All patients were reexamined 3–4 weeks after therapy. Persistence of BV occurred in 18/19 (95%) of women in phase I vs. 1/7 (14%) of women in phase II (*P* < 0.001). We concluded that, in women with coexistent clinically defined cervicitis and BV, the treatment of cervicitis does not result in a normalization of the vaginal flora.


Infectious Diseases in Obstetrics and Gynecology 3:119–122 (1995)
© 1995 Wiley-Liss, Inc.

### Pilot Study to Evaluate the Appropriate Management of Patients With Coexistent Bacterial Vaginosis and Cervicitis

**Jane R. Schwebke, Mary Beth Schulien, and Mark Zajackowski**

*Chicago Department of Health (J.R.S., M.B.S., M.Z.) and Medicine/Infectious Diseases, Northwestern University Medical School (J.R.S.), Chicago, IL*

### Abstract

**A pilot study was performed to obtain preliminary data regarding the need for targeted therapy of bacterial vaginosis (BV) when it accompanies clinically defined cervicitis. Specifically, women attending a sexually transmitted disease (STD) clinic with clinically defined BV and cervicitis were treated in the first phase of the study with doxycycline alone. In phase II, the patients received doxycycline and concomitant intravaginal metronidazole gel. All patients were reexamined 3–4 weeks after therapy. Persistence of BV occurred in 18/19 (95%) of women in phase I vs. 1/7 (14%) of women in phase II ($P < 0.001$). We concluded that, in women with coexistent clinically defined cervicitis and BV, the treatment of cervicitis does not result in a normalization of the vaginal flora.**
**© 1995 Wiley-Liss, Inc.**

Key words
STD, metronidazole, pilot study

The coexistence of bacterial vaginosis (BV) and cervicitis is frequently observed by clinicians and is well documented in the literature.[1–4] As many as 50% of the women attending sexually transmitted disease (STD) clinics with clinically defined cervicitis have BV.[3] Despite the frequent coexistence of these syndromes, no formal recommendations for the appropriate clinical management of patients with BV and cervicitis exist. In practice, many clinicians treat only the endocervical infections in such women. To begin to examine the management of BV in the setting of cervicitis and to determine the optimal therapy for patients with both syndromes, we conducted a pilot study of women with BV and cervicitis attending an inner-city STD clinic.

### SUBJECTS AND METHODS

Women presenting to the Chicago Department of Health STD Clinic with a clinical diagnosis of BV and concomitant cervicitis who were seen by the 2 study clinicians were eligible for the study. The study was approved by the Internal Review Board of the Chicago Department of Health and an informed consent was obtained from each participant. A patient was enrolled based on the investigator's assessment of her probable compliance and willingness to return for a follow-up visit. A patient was excluded if she had evidence of fungal or trichomonal vaginitis or if she was pregnant. The patients were examined according to a standardized clinical protocol. A speculum examination was performed, and the presence and amount of vaginal discharge were noted. Secretions in the vaginal vault were collected with a cotton-tipped swab. The pH of the secretions was determined using pH indicator strips (EM Science, Gibbstown, NJ). The vaginal fluid, diluted in saline, was examined microscopically and the presence of pseudohyphae,

**Address correspondence/reprint requests to Dr. Jane R. Schwebke, University of Alabama at Birmingham, 229 Tinsley Harrison Tower, 1900 University Boulevard, Birmingham, AL 35294-0006.**

Received June 19, 1995

Accepted August 17, 1995

Brief Report trichomonads, or clue cells was noted. A whiff test was performed using 10% KOH, and a vaginal smear was air-dried for later microscopic evaluation. The ectocervix was cleaned with large cotton swabs and the endocervix examined for signs of inflammation. An endocervical swab was obtained for Gram's stain and culture of *Neisseria gonorrhoeae*, which was processed using standard laboratory methods.[5] A second endocervical swab was collected for chlamydia testing (EIA or DNA probe). A bimanual examination was performed.

The clinical diagnosis of BV was based on Amsel's criteria and required the presence of 3 of the following findings: homogenous vaginal discharge, pH >4.5, positive whiff test, and clue cells.[6] Cervicitis was defined as a clinical observation of a yellow endocervical discharge. The presence of endocervical bleeding and increased numbers of endocervical neutrophils (>30 per oil immersion field within the endocervical mucus) was also recorded.[1,7,8]

Vaginal Gram's stains for the diagnosis of BV were interpreted in a blinded fashion using the scoring system of Nugent et al.[9] The quantitation of polymorphonuclear leukocytes on the endocervical smears was performed as described by Brunham et al.[7]

The study was divided into 2 phases. During the first phase, the patients were treated for cervicitis with doxycycline, 100 mg p.o. b.i.d. for 7 days, or doxycycline plus cefixime, 400 mg p.o. × 1 dose, depending on the results of the endocervical Gram's stain. No treatment for BV was provided at the initial visit. The patients were asked to refrain from unprotected sexual intercourse and to return for a second visit in 3–4 weeks. At visit 2, the patients were reexamined, and repeat vaginal wet preparations and smears and endocervical smears were obtained. The patients were questioned about interim sexual practices, symptoms, and douching. If BV was still present, the patients were treated at that time with oral metronidazole, 500 mg p.o. b.i.d. for 7 days.[10]

Phase II was conducted in the same manner except that the patients received treatment at visit 1 for their endocervical infections and for BV. To avoid multiple oral antibiotics, we prescribed topical metronidazole gel (one applicator intravaginally b.i.d. for 5 days).[11]

### RESULTS

Thirty-one patients were enrolled in this pilot study. Between November 1992 and September 1993, 23 women were enrolled for phase I. Nineteen (83%) of these patients returned for visit 2. Eight patients were enrolled in phase II between September 1993 and April 1994. One patient failed to return for visit 2. There were no significant differences in the study groups in age, race, symptomatology, or isolation rates of *N. gonorrhoeae* or *Chlamydia trachomatis* (Table 1).

**TABLE 1. Comparison of evaluable patients in phase I and II at enrollment**

| | Phase I (N = 19) | Phase II (N = 7) | *P* value |
|---|---|---|---|
| Mean age (years) | 26.7 | 30.0 | NS[a] |
| Race (African American) | 19/19 (100%) | 7/7 (100%) | NS |
| Symptoms at visit 1 | | | |
| Vaginal discharge | 15/19 (79%) | 5/7 (71%) | NS |
| Odor | 6/19 (32%) | 4/7 (57%) | NS |
| Gonorrhea or chlamydia at visit 1 | 2/19 (10%)[b,c] | 0/7 (0%) | NS |

[a]NS = not significant.
[b]Chlamydia test results unavailable for 2 patients.
[c]Gonorrhea test results unavailable for 1 patient.

At the follow-up visit, only one patient treated for cervicitis admitted to noncompliance with the doxycycline regimen. She had taken the medication for 5 of the 7 days. Four of 19 (21%) women in phase I and 1/7 (14%) women in phase II admitted to unprotected intercourse during the study period.

At the time of their return visits, 18/19 (95%) patients in phase I had persistent BV based on the clinical criteria[5] compared with only 1/7 (14%) metronidazole-treated patients ($P < 0.001$) (Table 2). For phase I, vaginal Gram's stains were available for 12 patients and all stains confirmed the clinical diagnosis of BV. No Gram's stain specimen was available to confirm the absence of BV in the single patient without clinical findings (this patient admitted to douching 2 days prior to her return visit). For phase II, vaginal Gram's stains were available to confirm the clinical findings for all patients. Although a persistence of clinical cervicitis was noted in 9/19 (47%) women in phase I vs. only 1/7 (14%) women in phase II, this difference was not statistically significant. The vaginal discharge symptoms resolved in 9/15 (60%) of phase I participants vs. 4/5 (80%) in phase II. A resolution of vaginal odor was noted by 3/6 (50%) in phase I vs. 4/4 (100%) in phase II. Neither finding was statistically significant.

**TABLE 2. Comparison of study populations at visit 2**

| | Phase I | Phase II | *P* value |
|---|---|---|---|
| Symptoms | | | |
| Resolution of vaginal discharge | 9/15 (60%) | 4/5 (80%) | NS[a] |
| Resolution of odor | 3/6 (50%) | 4/4 (100%) | NS |
| Signs | | | |
| Persistence of cervicitis | 9/19 (47%) | 1/7 (14%) | NS |
| Presence of BV | 18/19 (95%) | 1/7 (14%) | <0.001 |

[a]NS = not significant.

### DISCUSSION

BV is characterized by a dramatic shift in vaginal microbial ecology. The normal lactobacilli-predominant flora is replaced by increased concentrations of anaerobic and facultative anaerobic flora.[12] However, the pathogenesis, including whether the disappearance of the lactobacilli is a primary cause or a result, is unknown.[13,14] The syndrome of BV most commonly occurs among sexually active women and frequently coexists with other STDs.[1–4] Up to 50% of women attending STD clinics with mucopurulent cervicitis also have BV.[3] In fact, the frequent coexistence of cervicitis and BV has led some investigators to question whether local factors associated with cervical inflammation may predispose to alterations in the vaginal flora.[3,15]

At a practical level, the most appropriate treatment of women with both syndromes is an important consideration. Despite the fact that the successful treatment of BV requires an antimicrobial agent with excellent activity against anaerobes, many practitioners feel that the treatment of cervicitis alone may result in a resolution of BV in this setting. Also of concern is that the potential gastrointestinal side effects associated with doxycycline and metronidazole might lead to poor patient compliance. The current availability of efficacious topical preparations for treating BV obviates that concern,[11,16] although their added expense is a factor to be considered.

Our data suggest that treatment specific for both BV and cervicitis is necessary in order to promptly reestablish the normal vaginal ecology. Although the number of patients studied in this pilot study was small, the prospective nature of the study, combined with the strikingly different results between the 2 treatment groups, makes the results worthy of comment and suggests the need for further investigation. Further studies should include a larger number of patients from various health-care settings in a randomized, placebo-controlled study design.

Only 7% of the patients in our study had *N. gonorrhoeae* or *C. trachomatis* isolated as a cause of their cervicitis. Chlamydia testing was likely hampered by our lack of access to more sensitive diagnostic techniques such as cell culture or polymerase chain reaction (PCR); however, just like its male counterpart, nongonococcal urethritis, no pathogen is isolated from the majority of women with mucopurulent cervicitis.[17] Nonetheless, in a high-risk setting such as an STD clinic, the treatment of women with clinical signs of cervicitis is warranted.[17] Future studies with a larger study population should allow for meaningful analyses of subsets of patients with various etiologies of cervicitis. Of interest is that 86% of the women who were treated simultaneously with doxycycline and metronidazole had clinical resolution of their cervicitis at visit 2 compared with 53% of women who did not receive metronidazole at visit 1. Although not statistically significant, this finding raises the question of a possible role for anaerobes in the etiology of cervical inflammation.

In summary, we have shown that the simultaneous treatment of BV and cervicitis is effective in eradicating BV in women who present with both syndromes. The failure to specifically treat BV resulted in the persistence of abnormal vaginal flora. As more information becomes available on the potential complications of BV and the protective role of vaginal lactobacilli against vaginal and cervical infections, the prompt resolution of BV may assume even greater importance.

### ACKNOWLEDGMENTS

The authors thank the staff of the STD/HIV program at the Chicago Department of Health for their support and commitment to disease control.

### REFERENCES

1. Holmes KK: Lower genital tract infections in women: Cystitis, urethritis, vulvovaginitis, and cervicitis. In Holmes KK, Mårdh PA, Sparling PF, Wiesner PJ (eds): Sexually Transmitted Diseases. New York: McGraw-Hill, pp 527–545, 1990.

2. Paavonen J, Roberts PL, Stevens CE, et al.: Randomized treatment of mucopurulent cervicitis with doxycycline or amoxicillin. Am J Obstet Gynecol 161:128–135, 1989.
3. Paavonen J, Critchlow CW, DeRouen T, et al.: Etiology of cervical inflammation. Am J Obstet Gynecol 154:556–564, 1986.
4. Moi H: Prevalence of bacterial vaginosis and its association with genital infections, inflammation, and contraceptive methods in women attending sexually transmitted disease and primary health clinics. Int J STD AIDS 1:86–94, 1990.
5. Mårdh PA, Danielson D: *Neisseria gonorrhoeae.* In Holmes KK, Mårdh PA, Sparling PF, Wiesner PJ (eds): Sexually Transmitted Diseases. New York: McGraw-Hill, pp 903–916.
6. Amsel R, Totten PA, Spiegel CA, Chen KSC, Eschenbach DA, Holmes KK: Nonspecific vaginitis: Diagnostic criteria and microbial and epidemiological associations. Am J Med 74:14–22, 1983.
7. Brunham RC, Paavonen J, Stevens CE, et al.: Mucopurulent cervicitis—The ignored counterpart in women of urethritis in men. N Engl J Med 311:1–6, 1984.
8. Katz BP, Caine VA, Jones RB: Diagnosis of mucopurulent cervicitis among women at risk for *Chlamydia trachomatis* infection. Sex Transm Dis 16:103–106, 1989.
9. Nugent RP, Krohn MA, Hillier SL: Reliability of diagnosing bacterial vaginosis is improved by a standardized method of Gram stain interpretation. J Clin Microbiol 29:297–301, 1991.
10. Centers for Disease Control: 1993 sexually transmitted diseases treatment guidelines. MMWR 42:68–70, 1993.
11. Hillier SL, Lipinski C, Briselden AM, Eschenbach DA: Efficacy of intravaginal 0.75% metronidazole gel for the treatment of bacterial vaginosis. Obstet Gynecol 81:963–967, 1993.
12. Easmon CSF, Hay PE, Ison CA: Bacterial vaginosis: A diagnostic approach. Genitourin Med 68:134–138, 1992.
13. Sobel JD: Bacterial vaginosis—An ecologic mystery. Ann Intern Med 111:551–553, 1989.
14. Spiegel CA: New developments in the etiology and pathogenesis of bacterial vaginosis. Adv Exp Med Biol 224: 127–134, 1984.
15. Holmes KK, Chen KCS, Lipinski CM, Eschenbach DA: Vaginal redox potential in bacterial vaginosis (nonspecific vaginitis). J Infect Dis 152:379–382, 1985.
16. Hillier S, Krohn MA, Watts H, Wolner-Hanssen P, Eschenbach D: Microbiologic efficacy of intravaginal clindamycin cream for the treatment of bacterial vaginosis. Obstet Gynecol 76:407–413, 1990.
17. Centers for Disease Control and Prevention: 1993 sexually transmitted diseases treatment guidelines. MMWR 42(RR-14):49–50, 1993.
